# Residual atherosclerotic cardiovascular disease risk in statin-treated patients: mechanisms, therapeutic strategies, and future directions—a scoping review

**DOI:** 10.3389/fcvm.2026.1911458

**Published:** 2026-09-28

**Authors:** Muhammad Abdul Rehman, Malik Olatunde Oduoye, Davidson John Ozoemena, Edward Akosah Danso, Gabriel Chukwuka, Mary Abosede Odelade

**Affiliations:** 1 Department of Medicine, Nawaz Sharif Medical College (NSMC), Gujrat, Punjab, Pakistan; 2 Department of Research, The Medical Research Circle, Goma, Democratic Republic of Congo; 3 Department of Physiology, School of Medicine, Wayne State University, Michigan, MI, United States; 4 Department of Surgery, Korle-Bu Teaching Hospital, Accra, Ghana; 5 Department of Laboratory Services, Federal Medical Center, Abuja, Nigeria; 6 Department of Nursing and Health, State University of Southwest Bahia, Jequie, Brazil

**Keywords:** inflammation, lipid-lowering therapy, lipoprotein(a), low-density lipoprotein cholesterol, precision cardiovascular medicine, residual atherosclerotic cardiovascular disease risk, statins

## Abstract

**Background:**

Statins remain the cornerstone of pharmacological prevention for atherosclerotic cardiovascular disease (ASCVD) because of their proven ability to reduce low-density lipoprotein cholesterol (LDL-C) and major adverse cardiovascular events (MACE). However, many patients continue to experience ASCVD events despite achieving guideline-recommended LDL-C targets with maximally tolerated statin therapy, highlighting the persistence of residual ASCVD risk. This scoping review synthesized current evidence on residual ASCVD risk in statin-treated patients, focusing on its epidemiology, underlying mechanisms, clinical evidence, therapeutic strategies beyond statins, and barriers to optimal risk reduction.

**Methods:**

A scoping review was conducted in accordance with the Preferred Reporting Items for Systematic Reviews and Meta-Analyses Extension for Scoping Reviews (PRISMA-ScR) guidelines. Electronic databases were systematically searched, yielding 1,240 records. After duplicate removal and screening, 195 full-text articles were assessed for eligibility, and 35 studies were included in the final qualitative synthesis. The included evidence comprised randomized controlled trials, observational cohort studies, registry-based investigations, and systematic and narrative reviews.

**Results:**

The included studies were synthesized into five major themes: epidemiology of residual ASCVD risk, underlying mechanisms, clinical evidence from landmark trials and real-world studies, therapeutic strategies beyond statins, and challenges limiting effective risk reduction. Evidence consistently demonstrated that residual ASCVD risk persists despite substantial LDL-C reduction because of multiple interacting factors, including elevated triglyceride-rich remnant lipoproteins, lipoprotein(a), persistent inflammation, metabolic comorbidities, vascular aging, treatment-implementation barriers, and behavioral factors. Landmark clinical trials, including IMPROVE-IT, FOURIER, ODYSSEY OUTCOMES, REDUCE-IT, CANTOS, LoDoCo2, and CLEAR Outcomes, demonstrated incremental cardiovascular benefit from combination lipid-lowering and selected anti-inflammatory therapies, while emerging therapies targeting lipoprotein(a), IL-6 signaling, and RNA-based pathways offer promising strategies for further reducing residual ASCVD risk.

**Conclusion:**

Residual ASCVD risk remains a major challenge despite guideline-directed statin therapy. Effective risk reduction requires a comprehensive approach that extends beyond LDL-C lowering to include combination lipid-lowering therapy, inflammation-targeted interventions, lifestyle optimization, improved treatment adherence, and individualized risk assessment. Continued evaluation of emerging therapies and precision medicine approaches is essential to further reduce the global burden of ASCVD.

## Highlights

- Residual atherosclerotic cardiovascular disease (ASCVD) risk persists despite guideline-directed statin therapy and achievement of recommended LDL-C targets.
- Evidence from the past decade identifies lipoprotein(a), triglyceride-rich remnant lipoproteins, chronic inflammation, metabolic comorbidities, and treatment-implementation factors as major contributors to residual ASCVD risk.
- Landmark clinical trials support incremental cardiovascular benefit from combination lipid-lowering and selected anti-inflammatory therapies, although substantial residual risk remains.
- Comprehensive risk assessment should extend beyond LDL-C to incorporate biomarkers, inflammation, behavioral factors, and individualized risk profiling.
- Emerging therapies targeting lipoprotein(a), inflammatory pathways, and RNA- and gene-based mechanisms offer promising opportunities for precision cardiovascular prevention.

## 1 Introduction

Cardiovascular disease (CVD) remains the foremost cause of death and disability worldwide, claiming more than 19 million lives each year and accounting for nearly one-third of all global mortality (1). Although statin therapy has proven highly effective in lowering Low-Density Lipoprotein (LDL) cholesterol and reducing atherosclerotic cardiovascular events, a considerable proportion of patients continue to experience heart attacks and strokes (2). This persistent “residual risk” is associated with multiple factors beyond cholesterol control, including systemic inflammation, elevated triglycerides, metabolic disorders, and lifestyle-related risk factors (3).

The burden of CVD is shifting, with declining mortality in high-income countries (HICs) but rising prevalence in low- and middle-income (LMI) regions (4). Even among patients who achieve LDL targets with statins, elevated triglycerides, non-HDL cholesterol, and inflammatory markers remain common drivers of vascular injury (5). Co-morbid conditions and environmental exposures, such as obesity and air pollution, further compound the problem (3).

Residual cardiovascular risk in this review primarily refers to residual atherosclerotic cardiovascular disease (ASCVD) risk, defined as the continued occurrence of major adverse atherosclerotic cardiovascular events (MACE) despite guideline-directed statin therapy and appropriate LDL-C lowering (6). Throughout this review, ASCVD is used as the principal disease framework because statins primarily reduce risk through modification of atherosclerotic processes. Coronary artery disease (CAD), coronary heart disease (CHD), ischemic stroke, and peripheral arterial disease (PAD) are discussed as specific clinical manifestations of ASCVD, whereas the broader term cardiovascular disease (CVD) is reserved for general epidemiological contexts that include both atherosclerotic and non-atherosclerotic cardiovascular disorders. Likewise, the term atherosclerosis refers to the underlying pathological process rather than the clinical disease category.

In this review, optimal lipid lowering refers to the achievement of guideline-recommended LDL-C targets using maximally tolerated statin therapy, with or without additional evidence-based lipid-lowering therapies (e.g., ezetimibe or PCSK9 inhibitors) where clinically indicated (7). Accordingly, residual ASCVD risk is considered the risk that persists despite attainment of these recommended lipid-management goals, reflecting the contribution of non-LDL pathways and other biological, clinical, and treatment-related factors (8).

Statins reduce ASCVD risk primarily through inhibition of 3-hydroxy-3-methylglutaryl coenzyme A (HMG-CoA) reductase, the rate-limiting enzyme in hepatic cholesterol biosynthesis (9). Reduced intracellular cholesterol stimulates sterol regulatory element-binding protein (SREBP)-mediated upregulation of hepatic low-density lipoprotein receptors (LDLR), thereby increasing the clearance of circulating apolipoprotein B (apoB)-containing lipoproteins, particularly LDL particles (10). The resulting reduction in LDL-C is the principal mechanism through which statins lower the incidence of MACE, forming the basis for contemporary lipid-management guidelines and therapeutic targets (11).

Importantly, this LDL-C-centered pathway does not directly address all biological processes that contribute to ASCVD progression (11). Residual ASCVD risk therefore refers to the persistence of cardiovascular events despite appropriate statin therapy and LDL-C reduction, reflecting additional biological pathways, incomplete modification of atherogenic lipoprotein burden, inflammatory activity, metabolic dysfunction, plaque characteristics, treatment-implementation factors, and other determinants that extend beyond the primary pharmacological action of statins (6).

Statin dose-response generally follows the “rule of six,” whereby each doubling of the statin dose produces an additional reduction of approximately 6% in LDL-C, although the exact response varies among statins and individual patients (12).

Beyond lowering circulating LDL-C, statins promote favorable plaque remodeling (13). Imaging and pathological studies suggest reductions in lipid-rich plaque components, thickening of the fibrous cap, attenuation of vascular inflammation, decreased prevalence of high-risk plaque features, and gradual transformation toward more stable calcified plaques, thereby reducing plaque vulnerability rather than simply removing cholesterol from established lesions (14–16). They are effective for both primary prevention in individuals at high risk and secondary prevention in patients with established cardiovascular disease, consistently reducing all-cause mortality and major adverse atherosclerotic cardiovascular events (17). In addition to lipid-lowering, statins exert protective effects through anti-inflammatory activity, improved endothelial function, and reduced clot formation (17, 18). Because discontinuation can lead to a rebound in cholesterol levels and heightened cardiovascular risk, statins are generally prescribed for continuous, long-term use, underscoring their central role in modern cardiovascular care.

This ongoing risk of CVD highlights the limitations of statin monotherapy and underscores the need for broader preventive strategies. Emerging approaches include combination lipid-lowering therapies, anti-inflammatory agents, and integrated management of blood pressure, glucose, and lifestyle factors (19). Addressing residual ASCVD risk is therefore a critical challenge for modern cardiology, requiring both pharmacological innovation and holistic patient care. A ten-year review is justified because it captures the breadth of evidence needed to understand residual ASCVD risk in statin-treated patients. Over this period, large randomized trials, meta-analyses, and real-world registries have accumulated, providing a more reliable picture of how patients respond to therapy beyond LDL cholesterol reduction.

This timeframe also reflects demographic shifts, with declining mortality in high-income countries but rising burdens in low- and middle-income regions. It allows assessment of new therapies such as PCSK9 inhibitors, bempedoic acid, and anti-inflammatory agents. Synthesizing a decade of data, therefore, establishes a comprehensive rationale: to evaluate the limitations of statin monotherapy, identify persistent drivers of atherosclerotic cardiovascular events, and inform future strategies for integrated prevention and treatment. This study aims to critically evaluate the persistence of residual cardiovascular risk in patients treated with statins over the past decade.

## 2 Methodology

### 2.1 Study design

This scoping review was conducted according to the methodological framework for scoping reviews and reported in alignment with the Preferred Reporting Items for Systematic Reviews and Meta-Analyses Extension for Scoping Reviews (PRISMA-ScR) guidelines (20) focusing primarily on evidence published between January 2016 and June 2026. Earlier landmark cardiovascular trials were also considered, where necessary, to provide historical context and to establish the foundation for current approaches to residual cardiovascular risk management.

This review was intentionally conducted as a scoping review rather than a systematic review because the objective was to map and synthesize the breadth of evidence relating to residual ASCVD risk in statin-treated patients across multiple domains, including epidemiology, biological mechanisms, clinical trials, implementation barriers, and emerging therapeutic strategies. Given the heterogeneity of study designs, outcomes, and conceptual frameworks in this field, a scoping methodology was considered more appropriate for identifying knowledge gaps and summarizing evidence than a narrowly focused systematic review designed to answer a single clinical question.

### 2.2 Search strategy

We searched multiple databases, including PubMed, Embase, Cochrane Library, and Web of Science, using Boolean operators to refine our search strategy. The search string included terms such as *“residual cardiovascular risk” OR “persistent risk” AND (“statins” OR “HMG-CoA reductase inhibitors”) AND (“LDL cholesterol” OR “atherosclerosis” OR “PCSK9 inhibitors” OR “inflammation”).*

A comprehensive literature search was conducted to identify studies evaluating residual ASCVD risk in statin-treated patients. Four electronic databases PubMed/MEDLINE, Embase, Cochrane Library (CENTRAL), and Web of Science Core Collection were searched from January 1, 2016, to June 30, 2026. The final search was performed on July 2, 2026. The search strategy combined controlled vocabulary (Medical Subject Headings [MeSH] in PubMed and Emtree terms in Embase) with free-text keywords related to residual cardiovascular risk, statins, lipid-lowering therapies, inflammation, lipoprotein(a), and therapeutic interventions. Boolean operators (“AND”, “OR”) and database-specific syntax were applied as appropriate.

Only peer-reviewed articles published in English involving human participants were included. Eligible study designs comprised randomized controlled trials, prospective and retrospective cohort studies, case-control studies, systematic reviews, meta-analyses, clinical guidelines, and landmark consensus statements relevant to residual ASCVD risk. The reference lists of all included studies and relevant review articles were manually screened (backward citation tracking) to identify additional eligible publications. Forward citation tracking was also performed using Web of Science to identify recently published studies citing key landmark trials. The complete database-specific search strategies are provided in Supplementary Table S1.

### 2.3 Screening

From this search, 1,240 articles were initially identified. After removing duplicates, 1,050 remained. Title and abstract screening excluded 820 studies that were not relevant, leaving 230 for full-text review. Of these, 35 met the inclusion criteria and were incorporated into the final analysis. See Figure 1.

**Figure 1:**
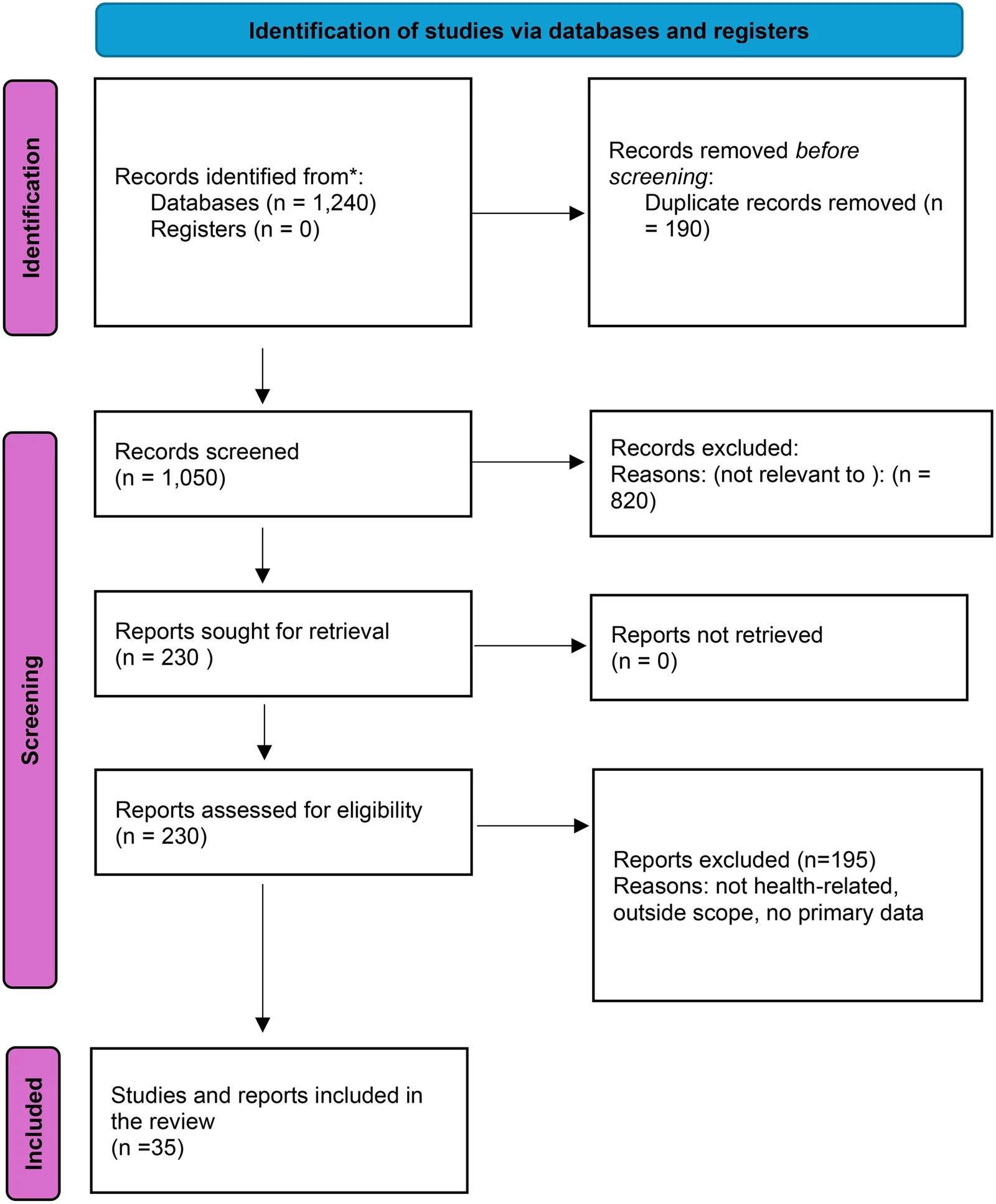
PRISMA-ScR flow diagram of study selection process.

### 2.4 Inclusion and exclusion criteria

Eligible studies included randomized controlled trials, observational studies, registries, systematic reviews, and relevant evidence syntheses that examined residual cardiovascular risk among statin-treated patients. Studies were included if they evaluated persistent atherosclerotic cardiovascular events, contributing mechanisms, risk factors, treatment strategies, or barriers to optimal risk reduction. Case reports, pilot studies, and non-peer-reviewed sources were excluded.

### 2.5 Data extraction

Data extraction focused on patient populations, interventions, outcomes such as myocardial infarction, stroke, and cardiovascular death, as well as mechanisms contributing to residual risk. The findings were synthesized thematically to highlight lipid-related, inflammatory, and comorbidity-driven pathways, as well as therapeutic strategies beyond statins.

## 3 Results

A total of 1,240 records were identified through database searching. After removal of duplicates, 1,050 articles remained for title and abstract screening. Following screening, 230 studies underwent full-text assessment, of which 35 studies met the eligibility criteria and were included in the final scoping review evidence synthesis. The included evidence comprised randomized controlled trials, observational cohort studies, registries, and systematic/narrative reviews evaluating residual cardiovascular risk among statin-treated patients who achieved LDL-C targets but continued to experience atherosclerotic cardiovascular events.

The included studies were grouped into five major themes: (3.1) epidemiology of residual cardiovascular risk, (3.2) mechanisms underlying persistent risk, (3.3) clinical evidence from major trials and real-world studies, (3.4) trial-specific framework, and (3.5) challenges limiting further cardiovascular risk reduction. Overall, the evidence demonstrated that although statin therapy substantially reduces LDL-C levels and atherosclerotic cardiovascular events, residual cardiovascular risk persists due to elevated triglycerides, lipoprotein(a), chronic inflammation, metabolic comorbidities, inadequate treatment adherence, and limitations of current risk stratification approaches. Detailed characteristics of all 35 included studies, including study design, population, primary findings, and corresponding reference numbers, are provided in Supplementary Table S2 to facilitate verification of the evidence synthesis.

The 35 included studies comprised randomized controlled trials (*n* = 14), observational cohort studies and registry-based investigations (*n* = 10), and systematic reviews, narrative reviews, and epidemiological analyses (*n* = 11). These studies were synthesized into five major themes: epidemiology of residual ASCVD risk (*n* = 9), underlying mechanisms of residual risk (*n* = 9), clinical evidence from landmark trials and real-world studies (*n* = 10), trials-specific framework (*n* = 12), and challenges limiting effective risk reduction (*n* = 6). Several landmark trials, including JUPITER, IMPROVE-IT, FOURIER, ODYSSEY OUTCOMES, REDUCE-IT, CANTOS, LoDoCo2, and CLEAR Outcomes, contributed evidence across multiple thematic domains because they informed both the mechanistic understanding of residual ASCVD risk and the evaluation of therapeutic interventions. Emerging studies evaluating lipoprotein(a)-targeted therapies (HORIZON and OCEAN(a)-Outcomes), oral PCSK9 inhibition (MK-0616), and IL-6 inhibition (RESCUE) were also incorporated to provide an updated perspective on future strategies for reducing residual ASCVD risk.

### 3.1 Epidemiology of residual risk

#### 3.1.1 Global burden of cardiovascular disease despite statin therapy

Across three studies, CVD continues to account for nearly one-third of all global deaths (21–23). The JACC analysis of 204 countries reported 19.2 million deaths in 2023 and 437 million DALYs, with almost 80% attributable to modifiable risk factors such as smoking, obesity, poor diet, and air pollution (23). Despite statin therapy, millions of patients still suffer recurrent events, underscoring the persistence of residual risk (22).

#### 3.1.2 Prevalence and residual risk

The *Lancet Global Health* 2025 article emphasized the “hidden burden” of CVD, noting that even patients considered low risk by traditional measures continue to experience significant morbidity (21). Statins reduce LDL cholesterol and lower event rates by 25%–37%**,** yet non-HDL cholesterol, triglycerides, and systemic inflammation remain common drivers of disease progression (21). A 2024 review highlighted that atherosclerotic CVD prevalence is rising sharply in LMICs**,** where access to preventive therapies is limited, and lifestyle risk factors are increasing (22).

#### 3.1.3 Regional trends

Mortality rates have declined in HICs due to better prevention and treatment, but the burden is shifting toward LMI regions**,** where CVD prevalence and mortality are climbing. This reflects inequities in healthcare access, limited use of combination therapies, and persistent exposure to environmental and lifestyle risks (1).

#### 3.1.4 Data from major trials

The evidence from major cardiovascular trials illustrates both the power and the limits of lipid-lowering therapy. The JUPITER trial showed that rosuvastatin significantly reduced LDL cholesterol and inflammatory markers in apparently healthy individuals with elevated hs-CRP, cutting the risk of heart attack, stroke, and cardiovascular death by nearly half (24). This demonstrated that statins confer benefits even in patients without high baseline LDL, highlighting their anti-inflammatory effects (24).

The IMPROVE-IT trial extended this evidence by testing the addition of ezetimibe to simvastatin in patients with acute coronary syndrome (ACS) (25). The combination achieved lower LDL levels than statin therapy alone. It produced a modest but statistically significant reduction in atherosclerotic cardiovascular events over seven years, confirming that incremental lipid lowering translates into clinical benefit (25). The FOURIER trial advanced the field further by evaluating evolocumab, a PCSK9 inhibitor, in patients already on statins. LDL cholesterol was reduced to an unprecedented level of around 30 mg/dL, and the risk of major atherosclerotic cardiovascular events fell by 15%. Importantly, safety was maintained even at very low LDL concentrations, proving that aggressive lipid lowering can achieve additional cardiovascular benefit while maintaining an acceptable safety profile (26).

#### 3.1.5 Patient populations most affected

A few studies demonstrate that residual cardiovascular risk despite statin therapy is concentrated in specific patient groups, with clear differences across age and gender (27–29). Adults with coronary artery disease (CAD) remain at high risk of recurrent events even when LDL cholesterol is controlled, largely due to persistent inflammation and elevated triglycerides (27). Patients with diabetes and metabolic syndrome are particularly vulnerable, as insulin resistance, obesity, and hypertension amplify vascular damage beyond lipid-lowering (28).

Age patterns reveal that middle-aged adults (40–65 years) carry the greatest burden, as cumulative exposure to risk factors manifests clinically during this period (30). Older adults (>65 years) experience the highest rates of recurrent atherosclerotic cardiovascular events, reflecting age-related vascular changes and multiple comorbidities (31). Meanwhile, younger adults (<40 years) are increasingly affected in LMICs, driven by rising obesity, diabetes, and tobacco use (32).

Gender differences are also evident. Men tend to experience an earlier onset of CVD and higher event rates, while women often present later but face unique risks, including underdiagnosis, atypical symptoms, and the impact of hormonal changes such as menopause (33). Both sexes remain vulnerable to residual risk, but the patterns of presentation and progression differ (33).

### 3.2 Mechanisms of residual risk

Residual cardiovascular risk persists through several overlapping biological mechanisms that statin therapy alone cannot fully address (Figure 2). Persistent LDL-C remains a problem in patients who fail to reach target thresholds, particularly those with familial hypercholesterolemia or statin intolerance (34). Every 1 mmol/L (≈39 mg/dL) LDL-C reduction lowers major vascular events by ∼22%, but residual risk remains when LDL-C is not fully suppressed (35). Beyond LDL, triglycerides and remnant lipoproteins contribute significantly to atherosclerosis. These particles deposit cholesterol into arterial walls and trigger inflammatory cascades, making them a major driver of residual risk (36). Patients with lipoprotein(a) >50 mg/dL had significantly higher event rates despite statin therapy (37). Moderately elevated lipoprotein(a) increased major adverse cardiac event (MACE) risk by ∼20%–30%, suggesting current thresholds underestimate risk (38).

**Figure 2:**
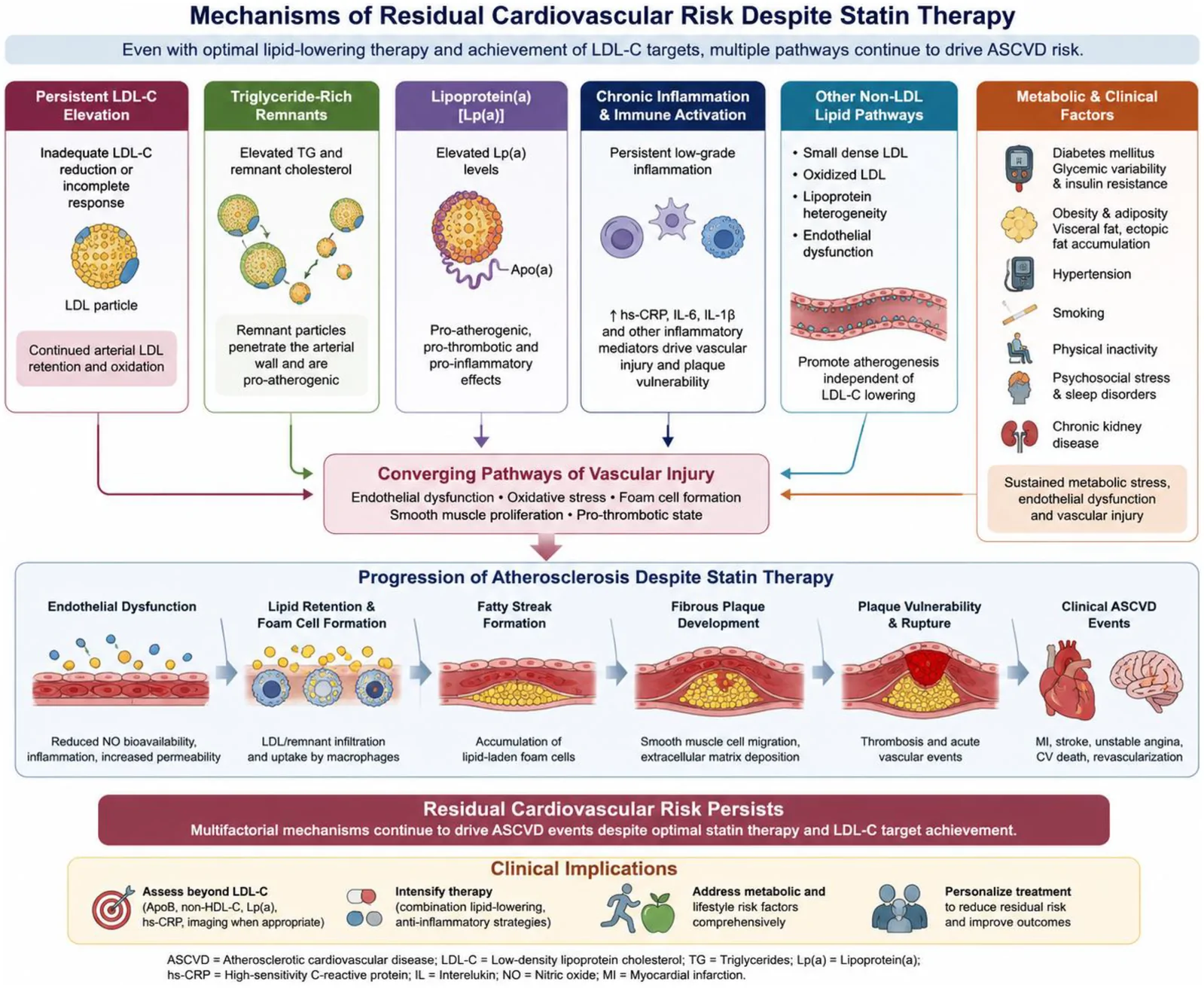
Mechanisms of residual atherosclerotic cardiovascular disease (ASCVD) risk despite statin therapy. Persistent apoB-containing lipoproteins, lipoprotein(a), triglyceride-rich remnant lipoproteins, chronic inflammation, and metabolic comorbidities converge to promote endothelial dysfunction, plaque progression, and recurrent ASCVD events despite optimal lipid-lowering therapy.

Inflammation remains central. Residual inflammatory risk persists even with optimal LDL-C lowering. A study confirmed that hs-CRP predicts events as strongly as LDL-C (39). Similarly, novel insights show that triglyceride-rich lipoproteins can reprogram T-cells, amplifying IL-6-mediated vascular inflammation (40).

Diabetes contributes to residual ASCVD risk through persistent metabolic dysfunction, endothelial injury, oxidative stress, and chronic inflammation, although these mechanisms interact with several other biological pathways (41). Age-related residual ASCVD risk may also reflect progressive loss of homeostatic reserve in genetically regulated pathways. LDL receptor-mediated clearance of apoB-containing lipoproteins may be adequate earlier in life but become less effective with aging, chronic inflammation, metabolic stress, increased PCSK9 activity, and cumulative vascular injury (42, 43). In this context, pharmacologic lipid-lowering therapy may become necessary because the functional capacity of endogenous lipid-regulatory pathways is no longer sufficient to maintain vascular homeostasis (44). This aging and genetic-reserve framework helps explain why residual risk often reflects both cumulative exposure to atherogenic particles and declining biological resilience despite LDL-C lowering.

Triglyceride-rich remnant lipoproteins are sufficiently small to penetrate the endothelial barrier and become retained within the arterial intima (45). Once retained, these particles deliver cholesterol directly to macrophages, promoting foam-cell formation and accelerating atherosclerotic plaque development (46). Unlike LDL particles, remnant lipoproteins may contain greater cholesterol content per particle, amplifying their atherogenic potential (47).

Lipoprotein(a) contributes to residual ASCVD risk through both atherogenic and prothrombotic mechanisms (48). In addition to transporting cholesterol, Lp(a) carries oxidized phospholipids that promote vascular inflammation (49). Structural homology between apolipoprotein(a) and plasminogen may interfere with fibrinolysis, thereby favoring thrombus formation and increasing cardiovascular risk (50).

The NLRP3 inflammasome–IL-1β–IL-6 signaling axis has emerged as a central pathway in residual inflammatory risk (51). Activation of the inflammasome promotes IL-1β release, which subsequently stimulates IL-6 production and hepatic synthesis of inflammatory mediators including C-reactive protein (52). Clinical benefit observed in anti-inflammatory trials supports the importance of this pathway in ASCVD progression (5).

Emerging evidence suggests that gut microbiota-derived metabolites, particularly trimethylamine N-oxide (TMAO), may contribute to residual ASCVD risk (53). TMAO has been associated with endothelial dysfunction, enhanced platelet activation, altered cholesterol metabolism, and increased inflammatory signaling, although the causal significance of these pathways remains under investigation (54).

### 3.3 Clinical evidence over the past decade

#### 3.3.1 Key findings from randomized controlled trials

Over the past decade, randomized controlled trials have provided compelling evidence that residual ASCVD risk persists even among statin-treated patients. These studies collectively demonstrate that while statins remain the cornerstone of lipid-lowering therapy, they do not eliminate the risk of recurrent atherosclerotic cardiovascular events.

#### 3.3.2 JUPITER trial

In apparently healthy individuals with elevated hs-CRP but normal LDL-C, rosuvastatin reduced major atherosclerotic cardiovascular events by 44%, confirming the benefit of statins in inflammation-driven risk. Yet, a substantial proportion of participants still experienced events, underscoring persistent inflammatory and metabolic pathways beyond LDL-C lowering (24).

#### 3.3.3 IMPROVE-IT trial

Among patients with ACS, adding ezetimibe to simvastatin achieved lower LDL-C (≈54 mg/dL) and a modest 2% absolute risk reduction in major events over seven years. This demonstrated that incremental LDL lowering yields benefit but cannot fully eliminate residual risk, highlighting the need for combination therapy (25).

#### 3.3.4 FOURIER trial

In patients with established atherosclerotic disease receiving statins, evolocumab (a PCSK9 inhibitor) reduced LDL-C to ≈30 mg/dL and lowered event rates by 15%. Despite achieving extremely low LDL levels, atherosclerotic cardiovascular events persisted, suggesting mechanisms such as inflammation, triglyceride-rich lipoproteins, and metabolic comorbidities continue to drive disease progression (26).

#### 3.3.5 ODYSSEY OUTCOMES AND CLEAR outcomes

Subsequent trials reinforced these findings. Alirocumab and bempedoic acid further reduced LDL-C and event rates, yet residual risk remained, particularly among patients with diabetes, obesity, and elevated hs-CRP (55).

#### 3.3.6 Observational studies and registries

Over the past decade, observational studies and registry data have provided crucial real-world confirmation that residual ASCVD risk persists in statin-treated patients. In the Reduction of Atherothrombosis for Continued Health (REACH) registry, among 296 patients (mean age 68 years, 75.7% men), 32.4% were classified as very high risk by the SMART score and 64.5% by the SMART-REACH model (17). Yet only 45.7% received high-intensity statins, and just 33.3% achieved LDL-C <55 mg/dL. Even fewer high-risk patients with diabetes were prescribed glucose-lowering drugs with proven cardiovascular benefit (17).

The European Action on Secondary and Primary Prevention by Intervention to Reduce Events (EUROASPIRE) surveys revealed poor lifestyle and risk factor control among coronary heart disease patients and those at high cardiovascular risk (56). Smoking, obesity, central obesity, and diabetes remained prevalent, with blood pressure and lipid control improving, but many patients still failing to reach targets. Glucose control showed no progress, and over 14 years, adverse lifestyle trends, including rising obesity and diabetes, were evident (56).

Large population-based cohorts add further mechanistic insight. In Denmark, an observational study of 327,347 patients found that remnant cholesterol increased all-cause mortality risk linearly, with a hazard ratio (HR) of 1.6 at 3 mmol/L compared to 0.9 mmol/L (57). Total cholesterol, LDL-C, non-HDL-C, and HDL-C all showed U-shaped associations with mortality, while triglycerides increased risk progressively (HR: 1.5 at 4.5 mmol/L) (57).

Similarly, an American study revealed a J-shaped association between non-HDL-C/HDL-C ratio (NHHR) and cardiovascular disease risk (58). A threshold effect was identified at NHHR 2.82, above which CVD risk increased by 21% per unit rise. Over seven years, cumulative remnant cholesterol exposure was strongly linked to cardiometabolic multimorbidity (CMM), with tertile 3 showing more than double the risk compared to tertile 1 (HR: 2.10) (58).

The China Health and Retirement Longitudinal Study provides strong evidence linking remnant cholesterol and inflammation to cardiometabolic multimorbidity (CMM) (59). In a cohort of 5,150 adults aged ≥45 years without baseline CMM, the cumulative remnant cholesterol–inflammation index (RCII) was tracked from 2011 to 2015. (59) During 7 years of follow-up, 294 participants developed CMM. Incidence rose steadily across RCII tertiles (3.2%, 5.2%, and 8.7%). Compared with the lowest tertile, those in the highest had more than double the risk (OR = 2.14; HR = 2.10) (59). Each 1-SD increase in RCII was associated with ∼33% higher odds and ∼32% higher risk of CMM. Dose–response analysis confirmed a linear relationship, and subgroup/sensitivity analyses supported the robustness of these findings (59).

Real-world outcomes also reveal disparities across age groups and gender. Older adults (>65 years) show the highest recurrence rates due to cumulative vascular damage and comorbidities, while men tend to experience earlier onset and higher event rates (60). Women, though often presenting later, face unique risks such as underdiagnosis and atypical symptoms (61). In LMICs, registry data confirm rising cardiovascular mortality, driven by limited access to advanced therapies and persistent lifestyle exposures (62).

#### 3.3.7 Subgroup analyses (age, sex, comorbidities)

Subgroup analyses reveal that residual cardiovascular risk despite statin therapy is unevenly distributed across age, sex, and comorbidities. Older adults (>65 years) consistently show the highest recurrence rates, reflecting cumulative vascular damage and multiple comorbidities (63). Middle-aged adults (40–65 years) carry a substantial burden as risk factors manifest clinically, while younger adults (<40 years) are increasingly affected in LMICs due to rising obesity, diabetes, and tobacco use (64).

Gender differences are also evident. Men tend to develop cardiovascular disease earlier and experience higher event rates, while women often present later with atypical symptoms and remain underdiagnosed (65). Post-menopausal women face additional risk as hormonal protection declines, underscoring the need for tailored strategies (66). Comorbidities such as diabetes, hypertension, obesity, and renal dysfunction amplify vascular injury beyond LDL-C control (67).

Elevated triglyceride-rich lipoproteins remain important contributors to residual ASCVD risk and are consistently associated with recurrent cardiovascular events across observational studies and randomized trials evaluating triglyceride-lowering interventions (68).

### 3.4 Trial-specific framework

#### 3.4.1 Established non-statin therapies

Ezetimibe typically provides an additional 15%–20% reduction in LDL-C when added to statin therapy, although individual responses vary considerably (69). Some patients achieve substantially greater LDL-C reductions, whereas others show more limited responses, reflecting heterogeneity in intestinal cholesterol absorption and overall lipid metabolism (70). The IMPROVE-IT trial (IMProved Reduction of Outcomes: Vytorin Efficacy International Trial) demonstrated that adding ezetimibe to simvastatin after acute coronary syndrome produced further LDL-C reduction and a modest but statistically significant reduction in cardiovascular events, supporting intensified lipid lowering beyond statin monotherapy (24).

The FOURIER trial (Further Cardiovascular Outcomes Research with PCSK9 Inhibition in Subjects with Elevated Risk) demonstrated that evolocumab, a PCSK9 inhibitor, produced substantial additional LDL-C lowering in statin-treated patients and significantly reduced major adverse cardiovascular events, although recurrent events continued to occur, indicating persistent residual ASCVD risk (26, 55). The ODYSSEY OUTCOMES trial showed that alirocumab reduced recurrent ischemic events in patients following acute coronary syndrome, particularly among individuals achieving substantial LDL-C reduction (26, 55).

#### 3.4.2 Emerging therapies

Bempedoic acid, an oral ATP citrate lyase inhibitor, lowers LDL-C by ∼20% alone and ∼35% with ezetimibe (71). The Cholesterol Lowering via Bempedoic Acid, an ACL-Inhibiting Regimen (CLEAR) Outcomes trial demonstrated that bempedoic acid reduced cardiovascular events among statin-intolerant patients, providing an additional treatment option for patients unable to tolerate intensive statin therapy (72). Inclisiran, a small interfering RNA therapy, reduces PCSK9 synthesis and provides durable ∼50% LDL-C lowering with twice-yearly dosing (73). ORION trial data confirm efficacy and tolerability; however, outcome trials are still ongoing (74).

#### 3.4.3 Anti-inflammatory approaches

Residual risk is also driven by inflammation. Canakinumab, an IL-1*β* inhibitor, reduced recurrent events in the Canakinumab Anti-inflammatory Therapy for Atherosclerotic Disease (CANTOS) trial, independent of LDL-C lowering, but safety and cost limit its use (75, 76). Colchicine, a low-cost anti-inflammatory, showed benefit in COLCOT and LoDoCo2, reducing recurrent ischemic events by targeting vascular inflammation (77).

#### 3.4.4 Omega-3 fatty acids and triglyceride-lowering agents

High triglycerides remain a major driver of residual risk. The Reduction of Cardiovascular Events with Icosapent Ethyl–Intervention Trial (REDUCE-IT trial) showed that icosapent ethyl significantly reduced cardiovascular events among statin-treated patients with elevated triglycerides, highlighting triglyceride-rich lipoproteins as an important therapeutic target beyond LDL-C lowering (78). Other triglyceride-lowering agents targeting ApoC-III and ANGPTL3 are under investigation, aiming to reduce remnant lipoprotein burden and inflammation (79).

#### 3.4.5 Lipoprotein(a)-targeted therapies

Lipoprotein(a) represents one of the most important currently undertreated pathways contributing to residual ASCVD risk (80). Pelacarsen, an antisense oligonucleotide targeting apolipoprotein(a), and olpasiran, a small interfering RNA therapy directed against *LPA* messenger RNA, have demonstrated substantial reductions in circulating Lp(a) concentrations in phase II studies (81–84).

#### 3.4.6 Oral PCSK9 inhibition

MK-0616 is an orally administered PCSK9 inhibitor that has demonstrated substantial reductions in LDL-C in early clinical studies (85–87). If long-term efficacy and outcome benefits are confirmed, oral PCSK9 inhibition may improve treatment accessibility and adherence compared with injectable therapies (88, 89).

#### 3.4.7 Emerging anti-inflammatory therapies

Beyond IL-1β inhibition, the IL-6 signaling pathway has emerged as an important target in residual inflammatory risk. Ziltivekimab, a monoclonal antibody directed against the IL-6 ligand, has demonstrated reductions in inflammatory biomarkers in high-risk populations (90). A study found that treatment with ziltivekimab produced substantial reductions in inflammatory biomarkers (91). Ziltivekimab was well tolerated, with no effect on the total cholesterol–to–HDL-cholesterol ratio. However, a small but statistically significant increase in triglyceride levels was noted in participants receiving 15 mg and 30 mg compared with placebo (91).

### 3.5 Challenges in risk reduction

Risk reduction faces three major challenges: limited adherence and statin intolerance, restricted access to novel therapies due to cost, and inadequate risk stratification for diverse patient populations.

#### 3.4.1 Patient adherence and statin intolerance

One of the most persistent challenges in lipid management is maintaining long-term statin use. A study shows that between 40% and 75% of patients stop taking statins within the first year of starting therapy (92). The reasons for discontinuation are varied, ranging from side effects to patient perceptions, but the impact is clear: poor adherence undermines LDL-C reduction and worsens cardiovascular outcomes (92, 93).

An additional challenge is distinguishing pharmacologic statin intolerance from nocebo-mediated or misattributed symptom burden. Placebo-controlled rechallenge studies, including SAMSON and StatinWISE, have shown that many symptoms attributed to statins are also reported during placebo exposure, suggesting that expectation and symptom attribution contribute substantially to perceived intolerance (94–96).

In real-world practice, non-adherence is often the leading cause of inadequate LDL-C lowering, leaving patients exposed to residual ASCVD risk despite the proven benefits of statins (97). Sustained adherence is therefore as critical as the drug's potency, since without consistent use, the protective effects of statin therapy cannot be fully realized (92).

Bradley *et al*. surveyed 5,693 adults eligible for statin therapy about their experiences and beliefs. Overall, 26.5% were not receiving treatment (98). Among these, 59.2% reported never being offered a statin, 10.1% declined therapy, and 30.7% had discontinued use (98). Women, Black adults, and those without insurance were more likely to report never being offered treatment (98).

Fear of side effects and perceived adverse reactions were the leading reasons for declining or stopping statins. Compared with current users, those who declined or discontinued were far less likely to view statins as safe or effective (98). Importantly, willingness to initiate or retry therapy remained high; nearly two-thirds of patients not on treatment expressed openness to starting or restarting a statin (98).

In a cohort of 22,340 Australians aged ≥65 years who began statin therapy between 2014 and 2015, adherence during the first year was suboptimal (99). More than half (55.1%) were nonadherent, and nearly half (44.7%) discontinued treatment. Rates were higher among general beneficiaries, who faced greater co-payments, compared with concessional beneficiaries (99).

Among concessional patients, older age (75–84 and ≥85 years) increased the likelihood of discontinuation compared with those aged 65–74 (99). Diabetes was linked to both nonadherence and discontinuation, while hypertension, angina, and heart failure were associated with better persistence. Anxiety predicted discontinuation, whereas polypharmacy surprisingly reduced the risk of stopping therapy (99). Statins initiated by general practitioners were also more likely to be discontinued. Similar predictors were observed in the general beneficiary group (99).

Contemporary information environments also influence adherence. Patient beliefs about cholesterol, statins, diet, and cardiovascular risk are increasingly shaped by social media, anecdotal experiences, and misinformation (100, 101). Effective residual-risk reduction therefore requires not only evidence-based therapy but also authentic, transparent, and patient-centered communication that addresses concerns about adverse effects while maintaining trust in the current evidence base (102).

#### 3.4.2 Cost and accessibility of novel therapies

Novel lipid-lowering agents, such as PCSK9 inhibitors and inclisiran, achieve substantial LDL-C reductions, but their high cost and limited reimbursement remain major barriers to widespread adoption. Reviews emphasize that these therapies are concentrated in high-income countries, while patients in LMI regions, where CVD burden is rising fastest, often lack access (103, 104). Even in wealthier settings, restrictive insurance coverage and co-payment requirements limit uptake, creating inequities in care (105). Oral agents like bempedoic acid offer convenience but remain more expensive than generic statins or ezetimibe, reinforcing affordability as a central challenge (106).

#### 3.4.3 Gaps in risk stratification and personalized medicine

Current risk assessment models remain heavily LDL-C focused, overlooking other drivers of residual risk such as triglycerides, lipoprotein(a), systemic inflammation, and metabolic comorbidities. Subgroup analyses show that older adults, women, and patients with diabetes or obesity are often under-recognized in traditional stratification tools (103, 105). Emerging approaches such as biomarker-based prediction (hs-CRP, Lp(a)), genetic profiling, and integration of lifestyle/environmental exposures offer promise for precision medicine (104). However, real-world adoption remains limited, with most guidelines still relying on LDL thresholds alone, leaving many high-risk patients undertreated or misclassified (106) (Table 1). Landmark Clinical Studies Informing Residual ASCVD Risk and Therapeutic Strategies in Statin-Treated Patients.

**Table 1:** Landmark studies informing residual ASCVD risk in statin-treated patients.

Evidence domain	Study (Year)	Population	Principal finding	Clinical implication
Epidemiology	ACC Global Burden (2025)	Global population	High ASCVD burden persists despite advances	Highlights continuing unmet prevention needs
Inflammation	JUPITER (2008)	Elevated hs-CRP	Rosuvastatin reduced events but inflammatory risk persisted	Supports inflammatory residual risk
Intensive LDL lowering	IMPROVE-IT (2015)	Post-ACS	Ezetimibe provided incremental benefit	LDL-C reduction alone does not eliminate risk
PCSK9 inhibition	FOURIER (2017)	Stable ASCVD	Evolocumab reduced MACE by ∼15%	Supports aggressive LDL-C lowering
PCSK9 inhibition	ODYSSEY OUTCOMES (2018)	Recent ACS	Alirocumab reduced recurrent events	Confirms benefit after ACS
Triglyceride reduction	REDUCE-IT (2019)	Elevated triglycerides	Icosapent ethyl reduced cardiovascular events	Residual triglyceride risk is clinically important
Inflammation	CANTOS	Prior MI	IL-1β inhibition lowered events independent of LDL-C	Confirms inflammatory pathway
Inflammation	LoDoCo2	Chronic coronary disease	Colchicine reduced recurrent events	Low-grade inflammation remains modifiable
Alternative lipid lowering	CLEAR Outcomes (2023)	Statin-intolerant patients	Bempedoic acid reduced cardiovascular events	Useful alternative for statin intolerance
Emerging therapies	HORIZON, OCEAN(a)-Outcomes, RESCUE	High-risk ASCVD	Lp(a)-lowering and IL-6 inhibition under evaluation	Represents next generation of residual-risk therapy

## 4 Discussion

This present study reinforces that statins remain the cornerstone of lipid-lowering therapy, but residual cardiovascular risk persists across diverse populations. Earlier landmark trials such as JUPITER and IMPROVE-IT established that statins reduce atherosclerotic cardiovascular events by 25%–37%, yet residual risk remained evident, particularly in patients with elevated hsCRP or diabetes (24, 25). The present study corroborates these observations, showing that inflammation and metabolic factors remain central drivers of risk even in optimally treated patients.

More recent trials, including FOURIER and ODYSSEY OUTCOMES, showed that PCSK9 inhibitors produce substantial additional LDL-C lowering and reduce event rates, but recurrent events persisted (26, 107). These findings support the benefit of intensive LDL-C

lowering, but that lowering alone does not eliminate residual ASCVD risk. Potential contributors include residual apoB-containing particle burden, Lp(a), remnant or TGrich lipoproteins, inflammatory activity, metabolic comorbidity, established plaque burden, environmental exposures, and treatment-implementation barriers. These contributors should be distinguished according to evidence type and causal status.

Observational registries such as EUROASPIRE previously reported poor lifestyle control among high-risk patients, with smoking, obesity, and diabetes remaining prevalent (108). The present review reinforces this, showing that lifestyle and comorbidity-driven risk factors continue to undermine long-term outcomes despite pharmacological advances.

While previous studies emphasized LDL-centric models, our study highlights growing recognition of biomarker-driven prediction and precision medicine approaches. Emerging evidence supports integrating hsCRP, Lp(a), and polygenic risk scores into risk stratification, moving beyond LDL thresholds alone (109). This represents a shift from prior paradigms toward more individualized prevention strategies.

The present review highlights that non-statin therapies provide incremental benefits. Ezetimibe offers modest LDL-C reduction when added to statins, as shown in IMPROVE-IT (25). PCSK9 inhibitors such as evolocumab and alirocumab achieve 50%–60% LDL-C lowering and ∼15% event reduction, proving their value in high-risk and statin-intolerant patients (26, 107). Emerging agents like bempedoic acid and inclisiran expand the therapeutic arsenal, with CLEAR Outcomes demonstrating benefit in statin-intolerant populations (110) (Table 2). Conceptual Framework of Residual ASCVD Risk After Optimal Lipid-Lowering Therapy.

**Table 2:** Conceptual framework of residual ASCVD risk after optimal lipid-lowering therapy.

Residual risk category	Major contributors	Clinical significance	Current management	Future directions
Biological residual risk	Elevated apoB-containing lipoproteins, remnant cholesterol, lipoprotein(a), persistent inflammation, diabetes mellitus, chronic kidney disease, plaque activity	Continued ASCVD events despite LDL-C goal attainment	High-intensity statins, ezetimibe, PCSK9 inhibitors, lifestyle modification	Lp(a)-targeted therapies, anti-inflammatory agents, gene-editing approaches
Measurement residual risk	Discordance between LDL-C and apoB/non-HDL-C, unmeasured Lp(a), inadequate assessment of plaque burden or inflammatory biomarkers	Underestimation of true cardiovascular risk	ApoB, non-HDL-C, hs-CRP, coronary imaging when appropriate	Multi-omics biomarkers and AI-assisted risk prediction
Treatment-implementation residual risk	Poor adherence, statin intolerance/nocebo effect, undertreatment, delayed treatment intensification, cost and access barriers	Failure to achieve guideline-recommended LDL-C targets	Shared decision-making, adherence interventions, combination therapy	Precision medicine and digital adherence monitoring
Behavioural/environmental residual risk	Smoking, unhealthy diet, obesity, physical inactivity, psychosocial stress, environmental exposures	Persistent vascular injury despite pharmacotherapy	Lifestyle modification and comprehensive risk-factor management	Population-level prevention strategies
Aging/genetic reserve residual risk	Vascular aging, cumulative endothelial injury, inherited susceptibility, reduced homeostatic reserve	Residual ASCVD risk increases with age despite lipid control	Individualized risk assessment	Genomic risk stratification
Currently undertreated pathways	Lipoprotein(a), remnant lipoproteins, inflammatory pathways, thrombosis	Important drivers of residual ASCVD risk lacking routine targeted therapy	Limited targeted treatment options	Pelacarsen, olpasiran, MK-0616, ziltivekimab, CRISPR-based therapies

Anti-inflammatory therapies, including canakinumab and colchicine, reduce recurrent ischemic events by targeting vascular inflammation, while icosapent ethyl addresses triglyceride-driven risk with significant event reduction in REDUCE-IT (111). Collectively, these therapies illustrate that combination strategies targeting lipids, inflammation, and triglycerides are essential to close the gap left by statin monotherapy. Despite progress with novel therapies, three persistent barriers continue to limit cardiovascular risk reduction. Adherence and statin intolerance remain critical, with up to 75% of patients discontinuing therapy within the first year. Fear of side effects and perceived intolerance drive this trend, though many patients express willingness to retry treatment (98, 110).

Cost and accessibility pose another challenge. Advanced agents such as PCSK9 inhibitors and inclisiran deliver substantial LDL-C reductions but remain prohibitively expensive, with limited reimbursement even in high-income countries. In low- and middle-income regions, affordability gaps exacerbate inequities where CVD burden is rising fastest (112, 113). Finally, gaps in risk stratification hinder effective prevention. Current models focus heavily on LDL-C, overlooking triglycerides, lipoprotein(a), inflammation, and comorbidities. Vulnerable subgroups, older adults, women, and patients with diabetes or obesity, are often undertreated or misclassified. Although biomarker-driven and genetic approaches show promise, their integration into routine practice remains limited (109).

This review found that recognizing the distinction between pharmacologic statin intolerance and nocebo-mediated or misattributed symptom burden is clinically important because residual ASCVD risk may increase when effective lipid-lowering therapy is unnecessarily discontinued (8, 114). Strategies such as shared decision-making, structured rechallenge, symptom tracking, lower-dose statin regimens combined with ezetimibe, and transparent communication about benefits and risks may help improve long-term adherence without dismissing patient-reported symptoms (69, 115).

### 4.1 Strengths of the study

This review has several strengths. First, it provides a comprehensive and up-to-date synthesis of the multifactorial mechanisms underlying residual ASCVD risk despite guideline-directed statin therapy, integrating evidence from epidemiological studies, randomized controlled trials, and real-world investigations. Second, the review adopts a structured conceptual framework that distinguishes biological, measurement, treatment-implementation, behavioural/environmental, aging/genetic, and emerging undertreated pathways contributing to residual risk, thereby offering a clinically relevant perspective for precision cardiovascular prevention. Third, it critically evaluates established and emerging therapeutic strategies beyond statins, including PCSK9 inhibitors, bempedoic acid, inclisiran, lipoprotein(a)-targeted therapies, anti-inflammatory agents, and gene-based approaches, highlighting future directions in residual risk management. Finally, the review follows the PRISMA-ScR reporting framework and incorporates evidence from recent landmark studies, enhancing the methodological transparency, clinical relevance, and applicability of its findings to contemporary cardiovascular practice.

### 4.2 Limitations of the study

This review has several limitations that should be acknowledged. First, as a scoping review, it was designed to map and synthesize the breadth of available evidence rather than quantitatively estimate treatment effects; consequently, no formal risk-of-bias assessment or meta-analysis was performed. Second, the review relied on published randomized controlled trials, observational studies, and systematic reviews, which may introduce publication bias. Third, heterogeneity in study populations, interventions, clinical endpoints, and follow-up durations limited direct comparisons across studies. Fourth, although a comprehensive search of major bibliographic databases was conducted, the review was restricted to English-language publications, and most included studies originated from high-income countries, which may limit the generalizability of the findings to low- and middle-income settings where the burden of atherosclerotic cardiovascular disease is rapidly increasing. Finally, while emerging therapies, including lipoprotein(a)-targeted agents, oral PCSK9 inhibitors, IL-6 inhibition, RNA-based therapeutics, and gene-editing approaches, show considerable promise, long-term efficacy, safety, cost-effectiveness, and implementation data remain limited. In addition, lifestyle, behavioural, and socioeconomic determinants were inconsistently reported across studies, restricting a comprehensive assessment of their contribution to residual ASCVD risk.

## 5 Future directions

Future directions in cardiovascular risk reduction emphasize biomarker-driven prediction, genetic and precision medicine, rational combination therapy, and careful evaluation of long-term safety and efficacy. These strategies aim to personalize care, expand therapeutic options, and ensure durable outcomes. A summary of emerging strategies and remaining challenges for addressing residual ASCVD risk is presented in Table 3.

**Table 3:** Future directions and emerging therapeutic approaches for reducing residual ASCVD risk beyond statin therapy.

Future direction	Current evidence/status	Potential clinical impact	Remaining challenges
Biomarker-driven risk prediction	hs-CRP, apoB, Lp(a), proteomics and metabolomics improve risk stratification beyond LDL-C	Earlier identification of high-risk patients	Validation and implementation
Precision medicine	Polygenic risk scores and genomic profiling	Personalized prevention	Limited clinical availability
Combination lipid-lowering therapy	Ezetimibe, PCSK9 inhibitors, inclisiran, bempedoic acid, oral PCSK9 inhibitors (MK-0616)	Greater LDL-C reduction	Cost and adherence
Lipoprotein(a)-targeted therapies	Pelacarsen, olpasiran, HORIZON and OCEAN(a)-Outcomes	Direct reduction of Lp(a)-mediated residual risk	Awaiting Phase III outcomes
Anti-inflammatory therapy	Colchicine, canakinumab, ziltivekimab (IL-6 inhibition)	Targets inflammation beyond LDL-C	Safety and patient selection
Triglyceride/remnant cholesterol lowering.	Icosapenta-ethyl, ApoC-III inhibitors, ANGPTL3 inhibitors	Addresses triglyceride-rich lipoprotein risk	More outcome trials needed
Lifestyle and metabolic optimization	Weight management, smoking cessation, exercise, diabetes and hypertension control	Addresses non-lipid drivers of residual risk	Long-term adherence
Gene-editing and RNA therapeutics	CRISPR-based therapies and RNA therapeutics under development	Potential durable risk reduction	Long-term safety, affordability, ethics

### 5.1 Biomarker-driven risk prediction

Emerging evidence shows that integrating proteomics, metabolomics, and multi-omics profiling can improve cardiovascular risk prediction beyond traditional LDL-C measures. Machine learning (ML) models have shown potential for improving cardiovascular risk prediction through integration of metabolomic and proteomic data, although further validation is required before routine clinical implementation (116).

### 5.2 Genetic and precision medicine approaches

Genomic testing and polygenic risk scores are increasingly used to identify individuals at heightened risk of cardiovascular disease. Precision medicine integrates genetic variants, lifestyle, and environmental exposures to tailor therapy (109). Advances in gene therapy and editing technologies also offer targeted interventions for monogenic disorders such as familial hypercholesterolemia (117, 118).

### 5.3 Combination therapy paradigms

Combination therapies targeting multiple pathways, such as beta-blockers with angiotensin-converting enzyme (ACE) inhibitors or lipid-lowering agents with anti-inflammatory drugs, are increasingly recognized for synergistic benefits. Evidence supports single-pill combinations to improve adherence and optimize blood pressure and lipid control (119, 120).

### 5.4 Long-term safety and efficacy considerations

Novel lipid-modulating therapies, including PCSK9 inhibitors, inclisiran, and ANGPTL4 antibodies, show durable LDL-C reductions with favorable safety profiles (103). However, long-term data are still emerging, particularly regarding off-target effects, immune responses, and sustained efficacy (121). Studies emphasize that extended follow-up is essential to confirm the long-term safety, detect off-target effects, and ensure the durability of RNA-based and gene-editing therapies (103, 106, 113).

### 5.5 Evidence hierarchy for emerging therapies

Future therapeutic strategies should be interpreted according to their current level of evidence. Established therapies with demonstrated reductions in major adverse cardiovascular events include statins, ezetimibe, PCSK9 monoclonal antibodies, bempedoic acid, icosapent ethyl, colchicine, and canakinumab in selected populations. In contrast, inclisiran has robust LDL-C-lowering efficacy, but cardiovascular outcome trials are still ongoing. Similarly, apo(a)-targeted therapies such as pelacarsen and olpasiran, ApoC-III inhibitors, ANGPTL3 inhibitors, and gene-editing approaches have shown substantial lipid-lowering effects. Still, definitive evidence for cardiovascular event reduction is pending. Distinguishing established outcome-proven therapies from emerging lipid-modifying technologies is essential when discussing future directions in residual ASCVD risk management.

Ongoing phase III outcome trials, including HORIZON (pelacarsen) and OCEAN(a)-Outcomes (olpasiran), are expected to determine whether selective Lp(a) lowering translates into reductions in major adverse cardiovascular events. Findings from the RESCUE program support further evaluation of IL-6-targeted approaches as potential strategies for reducing inflammation-mediated ASCVD risk (122).

## 6 Conclusion

Residual ASCVD risk remains a major challenge despite guideline-directed statin therapy and achievement of recommended LDL-C targets. This review demonstrates that persistent risk is multifactorial, arising from biological mechanisms such as elevated lipoprotein(a), triglyceride-rich remnant lipoproteins, persistent inflammation, metabolic comorbidities, and vascular aging, as well as measurement, treatment-implementation, and behavioral factors. Evidence from landmark clinical trials supports the use of combination lipid-lowering therapy and selected anti-inflammatory strategies to further reduce ASCVD events, although substantial residual risk persists in many patients.

Emerging therapies targeting lipoprotein(a), inflammatory pathways, and novel RNA- and gene-based approaches offer promising opportunities to address previously undertreated mechanisms of residual risk. Future cardiovascular prevention should therefore move beyond an LDL-C-centric approach toward comprehensive, personalized risk assessment and management that integrates advanced biomarkers, multimodal therapeutic strategies, lifestyle optimization, and equitable access to evidence-based care. Continued high-quality clinical research is needed to establish the long-term efficacy, safety, and cost-effectiveness of these emerging interventions and to translate precision cardiovascular medicine into routine clinical practice.

## Data Availability

The original contributions presented in the study are included in the article/Supplementary Material, further inquiries can be directed to the corresponding author/s.
