# Supplementary material for: Residual atherosclerotic cardiovascular disease risk in statin-treated patients: mechanisms, therapeutic strategies, and future directions—a scoping review
Source: Front Cardiovasc Med. 2026 Sep 28;13:1911458. doi: 10.3389/fcvm.2026.1911458 (PMC13655155; doi:10.3389/fcvm.2026.1911458)
Supplement: Supplementary file 1 [file Table1.docx]

**Supplementary Table S1. Complete Database Search Strategy**

| **Database** | **Search Strategy** |
| --- | --- |
| **PubMed (MEDLINE)** | **Search date:** July 2, 2026. **Period searched:** January 1, 2016–June 30, 2026. **Search string:** ("Hydroxymethylglutaryl-CoA Reductase Inhibitors"[Mesh] OR statin* OR atorvastatin OR rosuvastatin OR simvastatin OR pravastatin OR pitavastatin OR fluvastatin) AND ("Cardiovascular Diseases"[Mesh] OR "Atherosclerosis"[Mesh] OR ASCVD OR "atherosclerotic cardiovascular disease" OR coronary artery disease OR ischemic stroke OR peripheral arterial disease) AND ("Residual cardiovascular risk" OR "residual ASCVD risk" OR "residual risk" OR "major adverse cardiovascular events" OR MACE OR recurrence) AND ("Lipoprotein(a)"[Mesh] OR lipoprotein(a) OR Lp(a) OR apoB OR apolipoprotein B OR triglyceride* OR remnant lipoprotein* OR inflammation OR hs-CRP OR PCSK9 inhibitor* OR ezetimibe OR bempedoic acid OR inclisiran OR icosapent ethyl OR colchicine OR canakinumab). **Filters:** Humans; English; 2016–2026. |
| **Embase** | **Search date:** July 2, 2026. **Period searched:** January 1, 2014–June 30, 2025. **Search string:** ('statin'/exp OR statin*:ti,ab OR atorvastatin:ti,ab OR rosuvastatin:ti,ab OR simvastatin:ti,ab) AND ('atherosclerotic cardiovascular disease'/exp OR ASCVD:ti,ab OR coronary artery disease:ti,ab OR ischemic stroke:ti,ab) AND ('residual cardiovascular risk':ti,ab OR 'residual ASCVD risk':ti,ab OR residual risk:ti,ab OR MACE:ti,ab) AND ('lipoprotein(a)'/exp OR 'apolipoprotein B'/exp OR triglyceride*:ti,ab OR remnant lipoprotein*:ti,ab OR inflammation:ti,ab OR PCSK9 inhibitor*:ti,ab OR ezetimibe:ti,ab OR bempedoic acid:ti,ab OR inclisiran:ti,ab OR icosapent ethyl:ti,ab). **Limits:** Human; English; 2016–2026. |
| **Cochrane Library (CENTRAL)** | **Search date:** July 2, 2026. **Search string:** (statin* OR atorvastatin OR rosuvastatin OR simvastatin) AND ("residual cardiovascular risk" OR "residual ASCVD risk" OR MACE) AND (lipoprotein(a) OR apoB OR inflammation OR PCSK9 OR ezetimibe OR bempedoic acid OR inclisiran OR icosapent ethyl OR colchicine). Trials and systematic reviews published between January 2016 and June 2026 were considered. |
| **Web of Science Core Collection** | **Search date:** July 2, 2026. **Search string (Topic):** TS=((statin* OR atorvastatin OR rosuvastatin OR simvastatin) AND ("residual cardiovascular risk" OR "residual ASCVD risk" OR "major adverse cardiovascular events") AND (atherosclerosis OR ASCVD OR lipoprotein(a) OR apoB OR triglycerides OR remnant lipoproteins OR inflammation OR PCSK9 OR ezetimibe OR bempedoic acid OR inclisiran OR icosapent ethyl)). **Limits:** English language; Article or Review; 2016–2026. |

**Supplementary Table S2. Characteristics of the 35 Studies Included in the Scoping Review**

| **Ref. No.** | **Authors (Year)** | **Study design/type** | **Study focus / Population** | **Main outcomes / Key findings** |
| --- | --- | --- | --- | --- |
| 21 | Tang & Gao (2025) | Commentary/Review | Global cardiovascular disease burden | Demonstrated hidden burden of CVD despite conventional risk stratification; highlighted persistent residual ASCVD risk. |
| 22 | Xu et al. (2025) | Global epidemiological study | Global ASCVD burden attributable to lifestyle and metabolic risks | Rising ASCVD burden in LMICs driven by metabolic and lifestyle risk factors despite preventive therapies. |
| 23 | American College of Cardiology (2025) | Global epidemiological analysis | 204 countries and territories | Reported 19.2 million CVD deaths and emphasized modifiable risk factors accounting for most disease burden. |
| 24 | Ridker et al. (2008) (JUPITER) | Randomized controlled trial | Apparently healthy adults with elevated hs-CRP | Rosuvastatin reduced major cardiovascular events by 44%, but residual inflammatory risk remained. |
| 25 | Cannon et al. (2015) (IMPROVE-IT) | Randomized controlled trial | Acute coronary syndrome patients | Ezetimibe plus simvastatin further lowered LDL-C and modestly reduced cardiovascular events. |
| 26 | Sabatine et al. (2017) (FOURIER) | Randomized controlled trial | ASCVD patients receiving statins | Evolocumab achieved profound LDL-C reduction and 15% reduction in major cardiovascular events. |
| 27 | Galli et al. (2026) | Narrative review | Coronary artery disease | Reviewed biological mechanisms and therapeutic approaches for residual cardiovascular risk. |
| 28 | Kones (2013) | Narrative review | High-risk cardiovascular patients | Discussed undertreatment, poor adherence, inflammation, and metabolic contributors to residual risk. |
| 29 | Mach et al. (2024) | Expert consensus/report | Dyslipidaemia management | Proposed emerging lipid targets beyond LDL-C for reducing residual risk. |
| 30 | Ortega et al. (2024) | Population cohort study | Adults by age and sex | Described age-related differences in first cardiovascular manifestations. |
| 31 | Rodgers et al. (2019) | Review | Aging populations | Demonstrated aging as a major determinant of recurrent ASCVD events. |
| 32 | Strati et al. (2024) | Review | Early-onset type 2 diabetes | Linked diabetes to increased lifetime cardiovascular risk. |
| 33 | Mahowald et al. (2024) | Review | Men and women with CVD | Identified important sex disparities in cardiovascular presentation and outcomes. |
| 35 | Kang et al. (2025) | Cohort study | Patients undergoing PCI | Greater LDL-C reduction associated with fewer vascular events. |
| 36 | Jo (2025) | Review | Triglyceride-rich lipoproteins | Established remnant cholesterol as an important contributor to residual ASCVD risk. |
| 37 | Kaur et al. (2024) | Review | Lipoprotein(a) | Elevated Lp(a) independently increased cardiovascular event risk despite statin therapy. |
| 38 | Orfanos et al. (2023) | Systematic review | ASCVD patients | Quantified burden of elevated lipoprotein(a) and residual cardiovascular risk. |
| 39 | Nafari et al. (2022) | Prospective cohort | Community population | hs-CRP predicted cardiovascular events independently of LDL-C. |
| 40 | Xu et al. (2024) | Review | Triglyceride-rich lipoproteins | Demonstrated inflammatory effects of remnant lipoproteins. |
| 41 | Li et al. (2026) | Comprehensive review | Hypertension and CVD | Explained interaction between hypertension, inflammation, and vascular injury. |
| 45 | Chapman et al. (2025) | Expert review | Remnant lipoproteins | Summarized mechanisms linking remnant cholesterol to atherosclerosis. |
| 48 | Vinci et al. (2023) | Review | Lipoprotein(a) | Described atherogenic and thrombotic mechanisms of Lp(a). |
| 51 | Blevins et al. (2022) | Review | NLRP3 inflammasome pathway | Established IL-1β/IL-6 inflammatory axis as a therapeutic target. |
| 53 | Budoff et al. (2025) | Prospective cohort | Multi-Ethnic Study of Atherosclerosis | TMAO associated with increased incident cardiovascular events. |
| 17 | Siniawski et al. (2023) | Registry study (REACH) | Patients with established CVD | Demonstrated poor achievement of LDL-C targets despite statin therapy. |
| 56 | Kotseva (2017) (EUROASPIRE) | Registry survey | European CHD patients | Persistent obesity, smoking, and diabetes despite preventive care. |
| 57 | Engell et al. (2025) | Population cohort | 327,347 primary-care patients | Elevated remnant cholesterol independently predicted mortality. |
| 58 | Yang et al. (2025) | Cross-sectional study | US adults | Non-HDL-C/HDL-C ratio strongly associated with cardiovascular disease risk. |
| 59 | Zhang et al. (2026) | Prospective cohort | China Health and Retirement Longitudinal Study | Higher remnant cholesterol-inflammatory index predicted cardiometabolic multimorbidity. |
| 72 | CLEAR Outcomes Investigators (2023) | Randomized controlled trial | Statin-intolerant patients | Bempedoic acid reduced LDL-C and cardiovascular events. |
| 75 | Ridker et al. (CANTOS) | Randomized controlled trial | Previous MI with elevated hs-CRP | Canakinumab reduced recurrent cardiovascular events independent of LDL-C. |
| 77 | Nidorf et al. (LoDoCo2/COLCOT evidence) | Randomized controlled trial | Chronic coronary disease | Low-dose colchicine reduced recurrent ischemic events through anti-inflammatory effects. |
| 78 | Bhatt et al. (REDUCE-IT) | Randomized controlled trial | Statin-treated patients with elevated triglycerides | Icosapent ethyl significantly reduced major cardiovascular events. |
| 81–84 | Phase II Lp(a) studies (Pelacarsen/Olpasiran) | Phase II clinical trials | Patients with elevated Lp(a) | Marked reductions in circulating Lp(a); outcome trials ongoing. |
| 90–91 | RESCUE/Ziltivekimab studies | Phase II clinical trial | High inflammatory-risk patients | IL-6 inhibition substantially reduced inflammatory biomarkers with acceptable safety profile. |
